# Nervonic Acid Prevents HFD-Induced Metabolic Dysfunction and Is Associated with Gut Microbiota Remodeling

**DOI:** 10.3390/metabo16060399

**Published:** 2026-06-08

**Authors:** Cheng-Yu Jiang, Zong-Liang Huang, Jia-Ling Liu, Shao-Rong Cen, Rong-Min Lu, Cong-Bin Wei, Han-Yang Meng, Qi-Jiang Xu

**Affiliations:** 1 School of Medical Technology and Artificial Intelligence, Youjiang Medical University for Nationalities, Baise 533000, China; 01398@ymun.edu.cn (C.-Y.J.); 20241058426@stu.ymun.edu.cn (Z.-L.H.); 19178953164@163.com (C.-B.W.); 18778239697@163.com (H.-Y.M.); 2 Department of Food Inspection, Guangxi-Asean Food Inspection Center, Nanning 530022, China; jialingliu6@163.com; 3 Technology Innovation Center of Natural Fragrances and Flavors, State Administration for Market Regulation, Nanning 530022, China; 4 Bai Se Shi Bai Lin Lin Chang, Baise 533099, China; 13471676166@163.com; 5 Baise Forestry Research Institute, Baise 533021, China; lksycb2963985@163.com

**Keywords:** nervonic acid, obesity, hyperlipidemia, gut microbiota, gut microbiota association

## Abstract

Background: Obesity is closely associated with gut microbiota dysbiosis. Nervonic acid (NA; (15Z)-15-tetracosenoic acid) is a bioactive fatty acid with reported metabolic effects. This study aimed to investigate the associations between NA administration, gut microbiota composition changes, and host metabolic phenotypes in high-fat diet (HFD)-fed mice. Methods: C57BL/6J mice were fed an HFD for 12 weeks and concurrently administered NA at doses of 20, 40, and 60 mg/(kg·d) by gavage. Metabolic parameters, histopathological changes, and fecal microbiota composition (via 16S rRNA gene sequencing) were evaluated. Results: NA administration was associated with significantly attenuated HFD-induced increases in body weight and adipose tissue mass, as well as marked reductions in serum total cholesterol, triglycerides, and low-density lipoprotein cholesterol (all *p* < 0.05). Hepatic steatosis and adipose tissue inflammation were also attenuated. 16S rRNA gene sequencing revealed that NA was associated with the counteraction of HFD-induced gut microbiota dysbiosis, including alterations in α-diversity and community structure. NA was associated with higher relative abundances of taxa such as *Blautia*, *Oscillibacter*, *Faecalibaculum*, *Parabacteroides*, *Dubosiella*, and *Odoribacter* and lower relative abundances of *Lachnoclostridium*, *Mucispirillum*, and *Alistipes*. Within-group correlation analyses showed that genera with higher relative abundances were inversely associated with lipid parameters and adiposity, whereas genera with lower relative abundances correlated positively with these metabolic indicators. Conclusions: NA administration was associated with bidirectional changes in gut microbiota composition—the enrichment of certain taxa and the suppression of others—concomitant with the amelioration of HFD-induced metabolic dysfunction. These findings indicate correlations between NA, gut microbiota alterations, and improved metabolic phenotypes; however, causality remains to be established.

## 1. Introduction

Obesity, defined by a pathological excess accumulation of adipose tissue, is a chronic metabolic disorder reaching worldwide epidemic proportions and poses a major public health challenge [1,2]. This pathological state predisposes affected individuals to a wide spectrum of metabolic comorbidities, including dyslipidemia, insulin resistance, and non-alcoholic fatty liver disease (NAFLD), thereby heightening the risk of cardiovascular diseases, type 2 diabetes mellitus, and specific malignancies [3,4,5]. The pathogenesis of obesity involves intricate interactions between genetic, environmental, dietary, and lifestyle drivers [6,7]. Notably, gut microbiota dysbiosis is now recognized as a critical contributor to the development and exacerbation of obesity and its associated metabolic disorders [2].

The gut microbiota acts as a virtual metabolic organ, fermenting dietary fibers into short-chain fatty acids (SCFAs) and regulating host energy metabolism via the gut–liver and gut–brain axes [8,9,10]. Compositional and structural shifts in the gut microbiota can trigger dysregulation of whole-body energy metabolism and promote the development of obesity [11]. The causal link between microbiota and obesity has been verified via fecal microbiota transplantation (FMT) studies, which revealed that microbiota-deficient mice transplanted with obese-donor microbiota exhibit pronounced adiposity and metabolic dysfunction [12]. Overall, these results establish the gut microbiota as a promising therapeutic target for obesity intervention. However, current pharmacotherapies for obesity are often limited by adverse effects, motivating the search for safer, natural alternatives [13]. Given this, there is a pressing demand to develop natural, biocompatible candidate agents with excellent safety profiles.

Nervonic acid (NA; cis-15-tetracosenoic acid, C24:1Δ15) is a long-chain monounsaturated fatty acid initially identified for its high enrichment in the myelin sheaths of the mammalian nervous system. Regarding natural sources, nervonic acid is found in small amounts in deep-sea shark brain oil but is more abundant in specific plant oils [14]. The seed kernel oil of *Malania oleifera*, a tree species endemic to China, contains nervonic acid at levels as high as 55.70–67%, making it the plant resource with the highest known nervonic acid content to date [15], thus providing an ideal raw material for the sustainable development of nervonic acid. In recent years, nervonic acid has attracted growing research interest due to its pleiotropic biological functions [14]. Apart from its widely acknowledged neuroprotective effects, accumulating preclinical evidence has validated that NA exerts a marked regulatory role in host metabolic homeostasis and gut microbiota [16,17]. From a mechanistic perspective, existing studies have confirmed that supplemental NA can restore intracellular C24:1 sphingolipid homeostasis, trigger the PPARα/PGC-1α/SIRT1 signaling axis, and upregulate the β-oxidation process of fatty acids in the liver, which in turn mitigates obesity and its linked metabolic abnormalities [18]. Nevertheless, whether the metabolic benefits of NA against obesity are accompanied by alterations in gut microbiota composition remain unknown.

Thus, this work utilized a mouse model of obesity induced by a high-fat diet, together with 16S rRNA gene sequencing, to examine the impact of NA on intestinal microbial composition and its correlation with host metabolic traits.

## 2. Materials and Methods

### 2.1. Animal Experimental Design

Thirty-five specific-pathogen-free (SPF) male C57BL/6J mice (14–16 g, 4 weeks old) were obtained from Guangdong Vital River Laboratory Animal Technology Co., Ltd. (Foshan, China) (license No. SCXK(Yue)2022-0063) and housed at the SPF Animal Experimental Center of Youjiang Medical University for Nationalities. The experimental protocol was approved by the Laboratory Animal Use Ethics Committee of Youjiang Medical University for Nationalities (approval No. 2025070401). Mice were housed in polycarbonate cages (4 mice per cage) under a 12 h light/dark cycle (lights on at 08:00) at 22 ± 2 °C and 50–60% relative humidity with ad libitum access to food and water. Cages were randomly positioned in the housing room to avoid positional bias. Following a one-week acclimation period, mice were randomly assigned to five groups (*n* = 7 mice per group) using a random number table: Control group (normal chow diet, D12450B, Research Diets; 10% kcal fat, 70% kcal carbohydrate, 20% kcal protein; 3.9 kcal/g; see Supplementary Table S1), Model group (60% high-fat diet, D12492, Research Diets; 60% kcal fat, 20% kcal carbohydrate, 20% kcal protein; 5.2 kcal/g), low-dose nervonic acid group (LNA, high-fat diet + 20 mg/(kg·d) NA), medium-dose nervonic acid group (MNA, high-fat diet + 40 mg/(kg·d) NA), and high-dose nervonic acid group (HNA, high-fat diet + 60 mg/(kg·d) NA). Because the cages had a maximum capacity of 4 mice, the 7 mice in each group were allocated into two cages: one containing 4 mice and the other containing 3 mice. Different treatment groups were housed in separate cages to prevent cross-contamination of gut microbiota. Nervonic acid ((15Z)-15-tetracosenoic acid; CAS No. 506-37-6; purity 97%; batch No. I9EHRDHS; molecular formula C_24_H_46_O_2_; molecular weight 366.62) was purchased from Anji Nai Co., Ltd. (Anji, China). The NA solution was stored at −20 °C. NA was dissolved in corn oil and administered daily by oral gavage. The Control and Model groups received an equal volume of corn oil. Dose selection and the use of corn oil as a vehicle, as well as animal housing and intervention procedures, were performed as described previously [18,19].

Animal attrition and sample size balancing: Each group started with 7 mice (*n* = 7). During the first week of gavage, two mice in the Model group died due to accidental esophageal perforation (procedural complication). Necropsy revealed gastric perforation in one case and esophageal laceration with aspiration in the other, both consistent with gavage-related injury. No deaths were attributed to the high-fat diet or nervonic acid treatment. No other deaths occurred in any other group (Control, LNA, MNA, and HNA). To avoid statistical bias from unequal sample sizes (Model group, *n* = 5; other groups, *n* = 7), two mice from each of the other four groups were randomly excluded using a random number table. Exclusion was performed before any endpoint analysis, was blinded to outcome measures, and was not based on any health-related criteria (all excluded mice remained clinically healthy). Consequently, all five groups had a final sample size of *n* = 5 mice for all endpoint measurements (body weight, serum biochemistry, histology, and gut microbiota analysis). Data from the two deceased mice and the eight randomly excluded mice were completely excluded from all final analyses. Detailed attrition information (including animal ID, group, status, week of death/exclusion, cause, and relationship to treatment) is provided in Supplementary Table S2.

### 2.2. Sample Collection

At the end of the 12-week intervention, mice were deprived of food for 12 h and anesthetized with 2% isoflurane inhalation. Mice were euthanized by cervical dislocation under deep anesthesia. Humane endpoints were predefined as body weight loss > 20%, anorexia, or severe lethargy; no animals reached these endpoints. Blood samples were obtained via retro-orbital bleeding followed by centrifugation at 3000 rpm for 10 min at 4 °C; the separated serum was preserved at −80 °C pending measurement. The liver, epididymal white adipose tissue, perirenal fat, and fecal samples were quickly excised and weighed. Aliquots of liver and adipose specimens were immersed in 4% paraformaldehyde, whereas residual tissues were immediately frozen in liquid nitrogen and kept at −80 °C [20]. Sample collection and histological evaluation were performed by investigators blinded to group allocation.

### 2.3. Biochemical Analysis

Serum levels of triglycerides (TG), total cholesterol (TC), low-density lipoprotein cholesterol (LDL-C), and high-density lipoprotein cholesterol (HDL-C) were quantified using commercial standardized enzymatic assay kits. All kits were purchased from Elabscience (catalog Nos. E-BC-K261-M, E-BC-K109-M, E-BC-K205-M, and E-BC-K221-M). All detection procedures were strictly carried out following the manufacturer’s instructions.

### 2.4. Histopathological Analysis

Liver, epididymal adipose, and perirenal adipose tissue samples fixed in 4% paraformaldehyde were dehydrated, embedded in paraffin, and cut into 4–5 μm thick sections. The sections were then stained with hematoxylin and eosin (H&E) for histopathological observation. Tissue morphological changes were observed and photographed under an OLYMPUS BX45 light microscope (Olympus Corporation, Tokyo, Japan). For quantitative analysis, five random fields per section were captured at 200× magnification. Hepatic steatosis was quantified using ImageJ (version 2.0). NAFLD activity score (NAS) was assessed according to the Kleiner criteria, including steatosis (0–3), lobular inflammation (0–3), and hepatocellular ballooning (0–2); total NAS ranged from 0 to 8. Adipocyte cross-sectional area was measured using ImageJ with the polygon selection tool; 100 adipocytes per tissue were analyzed per mouse. All quantifications were performed by investigators blinded to group allocation. Staining and observation procedures were performed as described in a previous study [21].

### 2.5. Gut Microbiota Analysis

DNA extraction, PCR amplification, and sequencing. Fresh fecal samples were collected from each mouse at the end of the 12-week intervention. Total genomic DNA was extracted using the E.Z.N.A.™ Mag-Bind Soil DNA Kit (Omega, M5635-02, New York, NY, USA). The V3–V4 region was amplified using primers 341F (5′-CCTACGGGNGGCWGCAG-3′) and 806R (5′-GACTACHVGGGTATCTAATCC-3′) with 2× Hieff^®^ Robust PCR Master Mix (Yeasen, 10105ES03, Shanghai, China). PCR was conducted as follows: one round of 95 °C for 3 min; 5 cycles of 95 °C for 30 s, 45 °C for 30 s, and 72 °C for 30 s; 20 cycles of 95 °C for 30 s, 55 °C for 30 s, and 72 °C for 30 s; and one round of 72 °C for 5 min. Amplicons were purified using Hieff NGS™ DNA Selection Beads (Yeasen) and sequenced on the Illumina MiSeq (Sangon BioTech, Shanghai, China).

Sequence processing and OTU clustering. Paired-end reads were assembled using PEAR (v0.9.8). Low-quality bases were trimmed using a 10 bp sliding window (Q20 threshold); reads < 200 bp were discarded. After quality filtering, the sequencing depth per sample ranged from 65,938 to 124,031 reads (Supplementary Table S3). Effective tags were clustered into OTUs at ≥97% similarity using USEARCH (v11.0.667). Chimeric sequences and singleton OTUs were removed. Representative sequences were classified using the RDP Classifier against the RDP database (confidence threshold 0.7). DNA extraction blanks and PCR negative controls were included per batch.

Microbiome statistical analysis. Rarefaction curves were generated by subsampling reads at increasing depths (Supplementary Table S4). The curves plateaued by approximately 50,000 reads per sample. Based on the minimum sequencing depth (65,938 reads), all samples were rarefied to 50,000 reads to normalize the sequencing depth before diversity analyses. α-diversity indices (Shannon and ACE) were calculated using Mothur (v3.8.31) and compared using the Kruskal–Wallis test followed by Dunn’s post hoc test with Benjamini–Hochberg FDR correction (*q* < 0.05). Principal component analysis (PCA) was performed on OTU abundance data for unsupervised visualization of sample clustering. Principal coordinate analysis (PCoA) was performed based on Bray–Curtis distance to quantify compositional dissimilarity, and group separation was tested using PERMANOVA (adonis, 999 permutations), with *R*^2^ and *p*-values reported. Differential abundance was analyzed using ANCOM-BC as the primary method, complemented by LEfSe (Linear Discriminant Analysis Effect Size, LDA > 3.5) and the Kruskal–Wallis test with Dunn’s post hoc test (FDR-corrected, *q* < 0.05). Correlations between bacterial genera and metabolic parameters were assessed across all groups combined and within each intervention group separately using Spearman’s rank test with FDR correction (*q* < 0.05). Functional profiles were predicted using PICRUSt2 (v2.6.2); pathway abundances were compared using the Kruskal–Wallis test with FDR correction (*q* < 0.05).

### 2.6. Statistical Analysis

All experimental data are presented as the mean ± standard error of the mean (SEM) based on five mice per group (*n* = 5). Normality was assessed using the Shapiro–Wilk test and homogeneity of variance using Levene’s test. Based on these results, parametric (one-way ANOVA followed by Tukey’s HSD) or non-parametric (Kruskal–Wallis followed by Dunn’s post hoc) methods were selected for multiple-group comparisons. For longitudinal body weight data, a two-way repeated-measures ANOVA was performed using SPSS 27.0, with treatment as the between-subject factor and time as the within-subject factor. Mauchly’s test of sphericity was applied; when the sphericity assumption was violated, the Greenhouse–Geisser correction was used. Post hoc comparisons between groups at each time point were conducted using Bonferroni-adjusted pairwise tests. Two-group comparisons were conducted using Student’s *t*-test or Mann–Whitney U test as appropriate. All statistical analyses were performed using GraphPad Prism 10.1 and SPSS 27.0. A *p*-value < 0.05 was considered statistically significant.

## 3. Results

### 3.1. Nervonic Acid Intervention Attenuated Body Mass Gain and Fat Mass Deposition in HFD-Fed Mice

**Figure 1:**
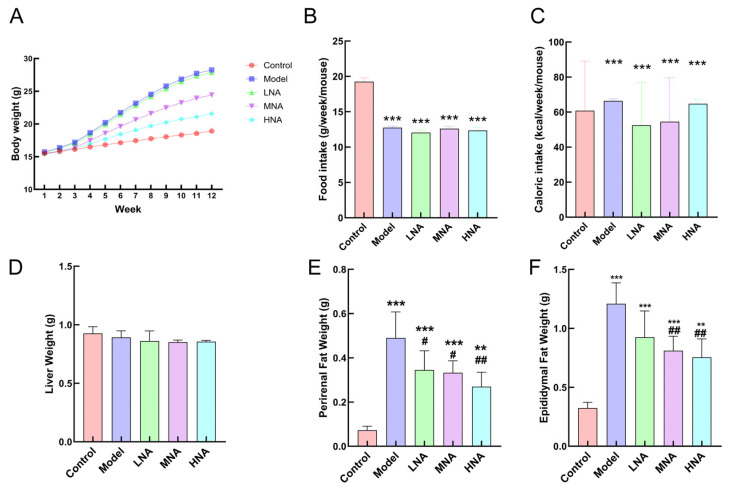
Effects of NA supplementation on body weight, caloric intake, and tissue weights in HFD-fed mice. (**A**) Body weight change over the 12-week supplementation period (two-way repeated-measures ANOVA). (**B**) Mean weekly food intake. (**C**) Weekly caloric intake (kcal/mouse/week), calculated from weekly food consumption and the energy density of each diet. (**D**) Liver weight. (**E**) Perirenal adipose tissue weight. (**F**) Epididymal adipose tissue weight. Data are presented as the mean ± SEM. Differences among groups were assessed using a one-way ANOVA followed by Tukey’s post hoc test (*n* = 5). ** *p* < 0.01, *** *p* < 0.001 vs. Control; # *p* < 0.05, ## *p* < 0.01 vs. Model.

In this preventive concurrent design, mice were fed a high-fat diet (HFD) and simultaneously supplemented with NA for 12 weeks. Longitudinal analysis of body weight using repeated-measures ANOVA revealed a significant group × time interaction (*p* = 0.001; full ANOVA results in Table S5). Post hoc comparisons showed that the body weight trajectory of the high-dose NA (HNA) group diverged significantly from that of the Model group from week 4 onward (Figure 1A). Importantly, weekly food intake and the calculated caloric intake did not differ significantly among the HFD-fed groups (one-way ANOVA, *p* > 0.05; Figure 1B,C), indicating that the attenuation of body weight gain was independent of reduced energy intake. At the end of the 12-week supplementation period, the weights of epididymal and perirenal adipose tissues were significantly lower in all NA-supplemented groups (LNA, MNA, and HNA) compared with the Model group (*p* < 0.05), whereas liver weight did not differ significantly among groups (Figure 1D–F).

### 3.2. Nervonic Acid Intervention Ameliorated Serum Lipid Profiles and Tissue Pathological Injury in HFD-Fed Mice

**Figure 2:**
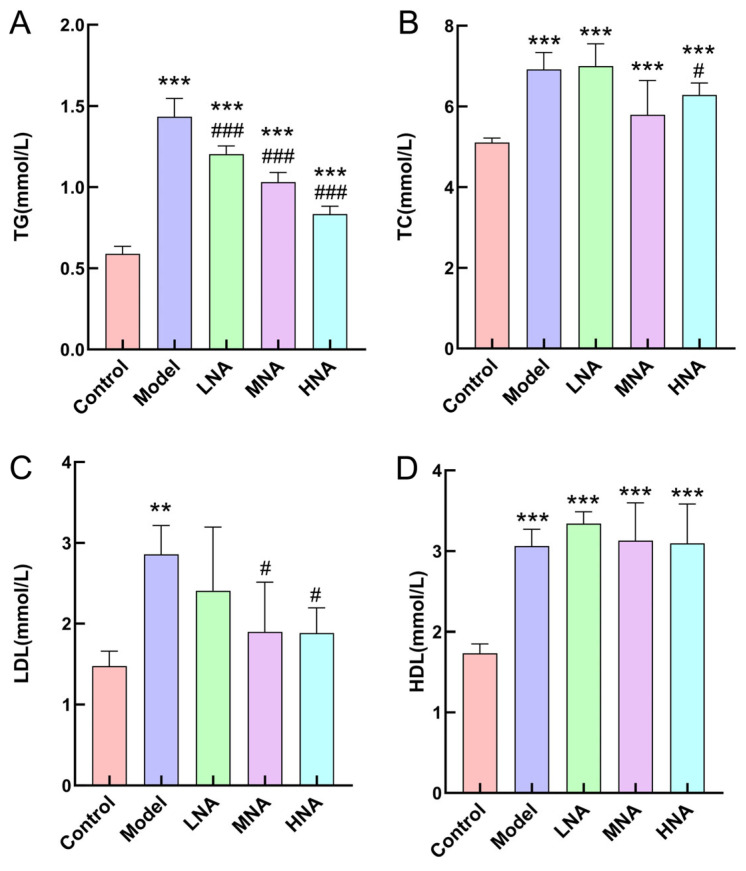
Ameliorative effects of NA on serum lipid profiles. (**A**–**D**) Serum levels of TG, TC, LDL-C, and HDL-C across all groups. Data are presented as mean ± SEM. Differences among groups were assessed using one-way ANOVA followed by Tukey’s post hoc test. (*n* = 5). ** *p* < 0.01, and *** *p* < 0.001 vs. Control; # *p* < 0.05, ### *p* < 0.001 vs. Model.

Serum biochemical analysis showed that the Model mice exhibited markedly higher levels of TG, TC, LDL-C, and HDL-C than the Control mice (*p* < 0.05). NA supplementation significantly ameliorated these dyslipidemic changes, resulting in obvious reductions in TG, TC, and LDL-C, whereas HDL-C levels showed no significant difference. The hypolipidemic effect was most prominent in HNA among all NA-treated cohorts (Figure 2A–D).

**Figure 3:**
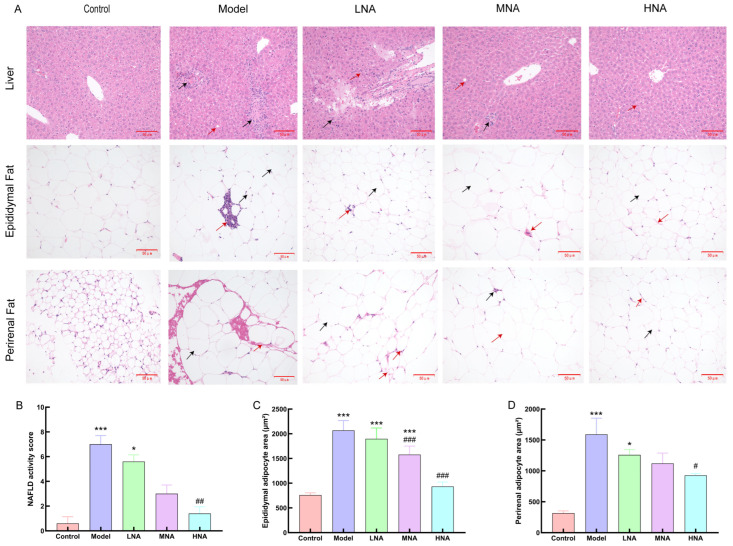
Ameliorative effects of nervonic acid on histopathological injury in obese mice. (**A**) Representative H&E-stained sections of liver, epididymal adipose tissue, and perirenal adipose tissue (200× magnification). Black arrows indicate lipid vacuoles; red arrows indicate inflammatory infiltration. (**B**) NAFLD activity score (NAS). (**C**) Mean adipocyte cross-sectional area in epididymal adipose tissue. (**D**) Mean adipocyte cross-sectional area in perirenal adipose tissue. Data are presented as mean ± SEM. Differences among groups were assessed using one-way ANOVA followed by Tukey’s post hoc test (*n* = 5). (Scale bar: 50 μm). * *p* < 0.05, *** *p* < 0.001 vs. Control; # *p* < 0.05, ## *p* < 0.01, and ### *p* < 0.001 vs. Model.

Histopathological evaluation revealed marked tissue damage in the Model mice (Figure 3A). In the liver, hepatocytes were arranged irregularly and exhibited severe cytoplasmic lipid vacuolation, focal necrosis, and inflammatory cell infiltration; consistent with these observations, the NAFLD activity score (NAS) was significantly higher in the Model group than in the Control group (Figure 3B). In epididymal and perirenal adipose tissues, adipocytes were markedly hypertrophic with heterogeneous sizes, and the mean adipocyte cross-sectional area in both fat depots was significantly larger in the Model group than in the Control group (Figure 3C,D). Following NA intervention, the pathological changes in the liver and adipose tissues were markedly attenuated: the NAS was significantly lower, and adipocyte hypertrophy in both epididymal and perirenal fat depots was significantly reduced compared with the Model group (*p* < 0.05; Figure 3B–D). Among all intervention groups, the HNA group exhibited the most pronounced histopathological improvement.

### 3.3. Nervonic Acid Intervention Was Associated with Altered Gut Microbiota Structure in HFD-Fed Mice

**Figure 4:**
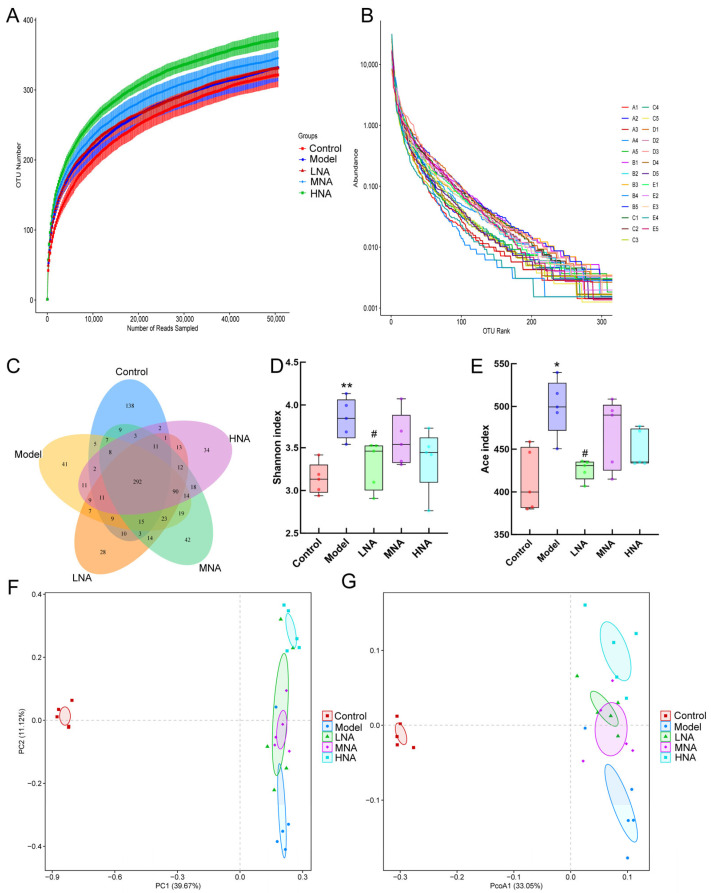
Effects of NA on gut microbiota in HFD-induced obese mice. (**A**) Rarefaction curves. (**B**) Rank–abundance curves. (**C**) Flower plot calculated at OTU level. (**D**,**E**) α-diversity indices. (**F**) PCA calculated at OTU level. (**G**) PCoA calculated at OTU level. Data are presented as mean ± SEM. Differences among groups were assessed using one-way ANOVA followed by Tukey’s post hoc test. (*n* = 5). * *p* < 0.05, ** *p* < 0.01 vs. Control; # *p* < 0.05 vs. Model.

Rarefaction curves plateaued across all groups with increasing sequencing reads, confirming an adequate sequencing depth to capture the majority of microbial diversity (Figure 4A). Rank–abundance curves revealed that, relative to the Control, the Model mice displayed a shorter horizontal span and steeper slope, indicating reduced OTU richness and increased dominance after HFD feeding. Following the NA intervention, the curve span and slope of HNA were improved, approaching Control levels, suggesting restoration of OTU evenness (Figure 4B). The distribution of shared and unique OTUs among groups is presented in Figure 4C.

α-diversity analysis demonstrated that Shannon and ACE indices were significantly higher in the Model group than in the Control (*p* < 0.05), indicating that HFD markedly altered gut microbiota diversity and richness. After NA administration, these α-diversity indices were lower in the NA-treated groups compared to the Model group, with the most pronounced difference observed in the LNA group (*p* < 0.05) (Figure 4D,E). To evaluate overall structural differences in gut microbiota across groups, principal component analysis (PCA) and principal coordinate analysis (PCoA) based on Bray–Curtis distance were performed (Figure 4F,G). Samples from the Control and Model groups showed distinct clustering separation, indicating that the HFD significantly reshaped the global gut microbiota structure. After the NA intervention, the clustering distribution of all supplementation groups deviated from the Model group, with HNA exhibiting a closer clustering distance to the Control and stronger intra-group aggregation. PERMANOVA confirmed significant differences in microbial community composition among all five groups (*R*^2^ = 0.566, *p* = 0.001). Pairwise comparisons showed that the Control group differed significantly from the Model group and all NA-treated groups (all *p* < 0.01). Among the NA-treated groups, HNA differed significantly from the Model group (*p* = 0.009), whereas MNA did not (*p* = 0.075) (Table 1).

### 3.4. NA Intervention Was Associated with Altered Gut Microbiota Composition in HFD-Fed Mice

**Figure 5:**
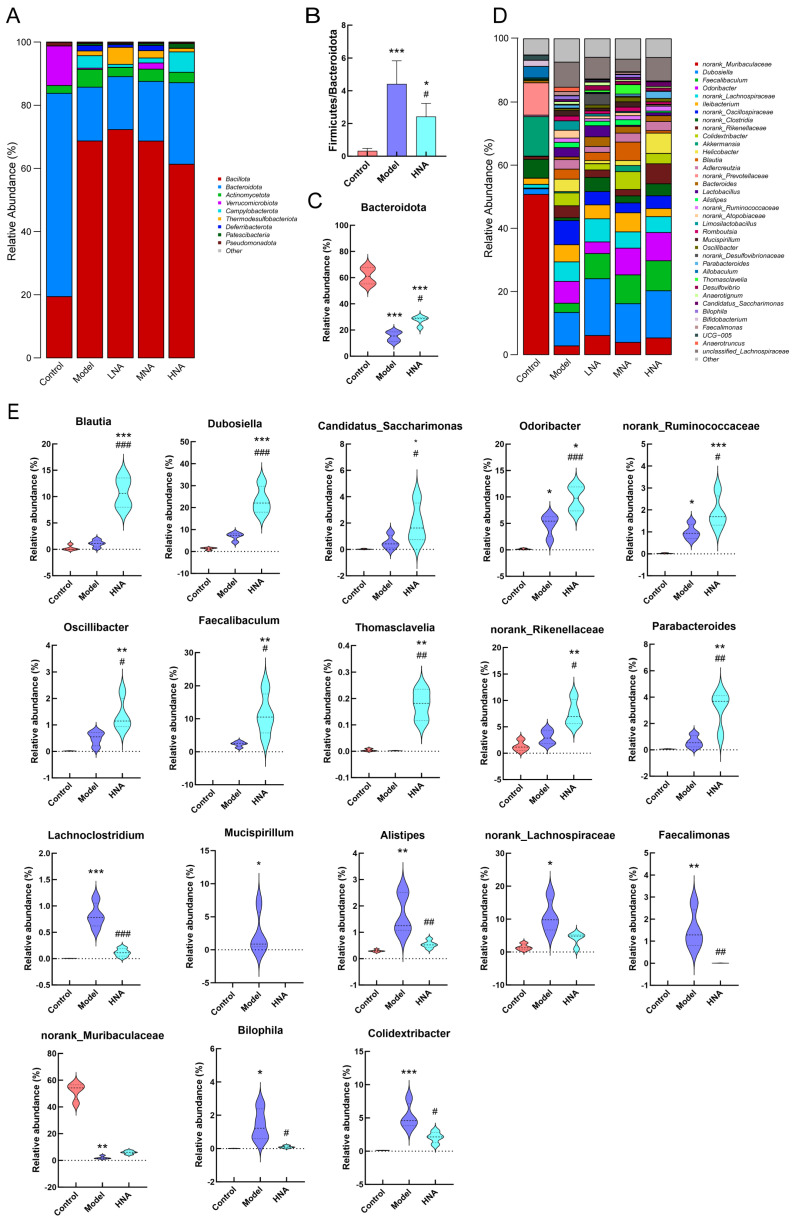
Effects of NA on gut microbiota composition in mice with HFD-induced obesity. (**A**) Relative abundance of gut microbiota at the phylum level. (**B**) Firmicutes/Bacteroidota (F/B) ratio. (**C**) Relative abundance of Bacteroidota. (**D**) Relative abundance at genus level. (**E**) Statistical analysis of relative abundance differences in representative bacterial genera. Data are presented as mean ± SEM. Differences among groups were assessed using one-way ANOVA followed by Tukey’s post hoc test or Kruskal–Wallis test. (*n* = 5). * *p* < 0.05, ** *p* < 0.01, and *** *p* < 0.001 vs. Control; # *p* < 0.05, ## *p* < 0.01, and ### *p* < 0.001 vs. Model.

To further explore the association between the NA intervention and intestinal microbial composition, we examined the microbial communities at both the phylum and genus levels. The relative abundances of gut microbiota at the phylum level across different groups are presented in Figure 5A. Compared with the Control, the Model group exhibited a significant reduction in Bacteroidota abundance (*p* < 0.05) and a concurrent increase in Firmicutes abundance, leading to a marked rise in the Firmicutes/Bacteroidota (F/B) ratio. In the NA intervention groups, Bacteroidota abundance was significantly higher than in the Model group, and the F/B ratio was correspondingly lower; these differences were most pronounced in HNA (Figure 5B,C).

At the genus level, HFD induced substantial alterations in gut microbiota composition (Figure 5D). Dominant genera in each group included *Mucispirillum*, *Anaerotruncus*, *norank_Muribaculaceae*, *Dubosiella*, *Faecalibaculum*, *Odoribacter*, and *norank_Lachnospiraceae*. Supplementary Figure S1 lists the top 25 genera with significant abundance differences between the Control and Model groups, as well as between the Model group and each NA intervention group. LEfSe analysis was further conducted to identify bacterial taxa with significantly differential abundances (LDA score > 3.5, *p* < 0.05) (Supplementary Figure S2). At the genus level, the Control was characterized by enrichment of *norank_Muribaculaceae*, *Akkermansia*, *norank_Prevotellaceae*, and *Allobaculum* (nine taxa); the Model group was enriched with *Mucispirillum*, *Anaerotruncus*, and *norank_Oscillospiraceae* (twelve taxa). Each NA intervention group displayed distinct microbiota enrichment patterns: LNA showed enrichment of *Dubosiella*, *norank_Lachnospiraceae*, *Lactobacillus*, *norank_Desulfovibrionaceae*, *Desulfovibrio*, and *Anaerotignum* (six taxa); MNA was enriched with *Blautia*, *Colidextribacter*, *Thomasclavelia*, *Alistipes*, *Oscillibacter*, and GCA-900066575 (six taxa); and HNA exhibited enrichment of *Faecalibaculum*, *Odoribacter*, *Helicobacter*, *norank_Rikenellaceae*, *Parabacteroides*, *norank_Ruminococcaceae*, and *Candidatus_Saccharimonas* (seven taxa). The relative abundances of these representative differential genera across groups are presented in Figure 5E.

### 3.5. Correlation Analysis of Differential Microbiota with Host Metabolic Indexes and Functional Prediction

**Figure 6:**
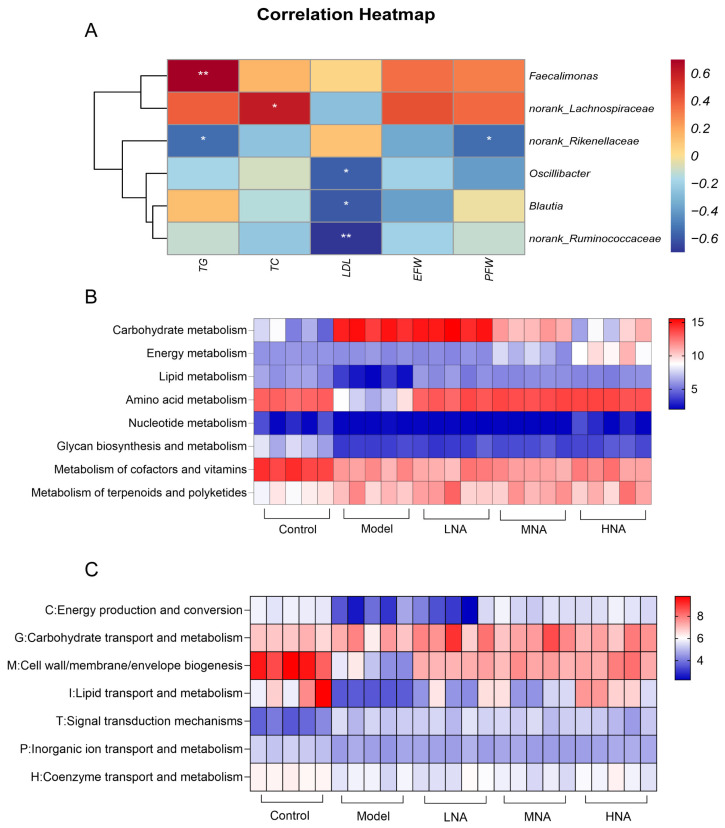
Correlation analysis and functional prediction of nervonic acid-mediated gut microbiota in ameliorating obesity-associated hyperlipidemia. (**A**) Correlation heatmap of dominant bacterial genera (**B**) KEGG pathway prediction (**C**) COG functional prediction. Data are presented as mean ± SEM (*n* = 5). * *p* < 0.05, ** *p* < 0.01.

Spearman correlation analysis was implemented to explore the associations between shifts in gut microbial community composition and host metabolic characteristics. As illustrated in Figure 6A, numerous discriminatory genera demonstrated statistically significant correlations with circulating lipid profiles and adipose tissue mass. Specifically, the relative abundance of *norank_Lachnospiraceae* displayed a positive correlation with serum TC concentrations (*p* < 0.05), while *Faecalimonas* exhibited a positive association with TG levels (*p* < 0.05). Conversely, several taxa enriched following the NA intervention showed negative correlations with metabolic markers: the relative abundances of *Oscillibacter*, *norank_Ruminococcaceae*, and *Blautia* were inversely correlated with LDL-C levels (*p* < 0.05); *norank_Rikenellaceae* was negatively associated with TG levels and perirenal fat weight (PFW) (*p* < 0.05). The direction of these associations is consistent with the metabolic improvements in the NA-treated groups, but further investigation is required to determine whether these taxa contribute directly to the observed benefits.

To explore differences in the inferred metabolic potential of the gut microbiota among groups, KEGG and COG functional categories were predicted using PICRUSt2 based on 16S rRNA gene sequencing data. KEGG pathway inference showed that, compared with the Control group, the inferred abundance of carbohydrate metabolism-related functions was elevated in the Model group and gradually decreased following intervention with different doses of nervonic acid (Figure 6B), whereas the inferred abundances of lipid metabolism and nucleotide metabolism were lower in the Model group; after NA intervention, lipid metabolism inferences modestly increased, while nucleotide metabolism inferences remained largely unchanged. The inferred abundances of amino acid metabolism and cofactor and vitamin metabolism were maintained at relatively high levels across all groups, with minor intergroup differences. COG functional inference yielded trends largely consistent with the KEGG analysis (Figure 6C): the inferred abundance of carbohydrate transport and metabolism was upregulated in the Model group and remained high after the NA intervention; cell wall/membrane biogenesis and lipid transport and metabolism inferences were downregulated in the Model group and gradually increased with an increasing NA dose; and in contrast, energy production and conversion inferences remained low in both the Model group and all NA-treated groups. Overall, marked differences in the inferred abundances of multiple microbial metabolic functions were observed among groups, with carbohydrate- and lipid-related functions showing the most pronounced changes. It should be noted that these results reflect only functional potential predicted from genomic sequences and do not represent direct measurements of actual pathway activities.

## 4. Discussion

### 4.1. Attenuating Effects of NA on HFD-Induced Metabolic Changes

In this study, mice receiving NA concurrently with an HFD showed attenuated body weight gain and reduced adipose tissue deposition compared with those receiving an HFD alone. From week 4 onward, body weight gain in the HNA group was notably lower than that in the Model group, and this difference persisted throughout the experimental period. The lower epididymal and perirenal adipose tissue weights in NA intervention groups further indicated that NA administration was associated with reduced excessive fat accumulation under HFD conditions. Importantly, weekly food intake and calculated caloric intake did not differ significantly among the HFD-fed groups, indicating that the attenuation of body weight gain was independent of reduced energy intake. This observation suggests that NA may attenuate HFD-induced weight gain through mechanisms involving energy expenditure or lipid metabolism rather than appetite suppression.

These findings are consistent with previous studies. Using an animal model, Keppley et al. [18] reported that isocaloric diets enriched with NA were associated with alleviated HFD-induced weight gain, alongside restored C24:1 sphingolipid levels and activation of the PPARα/PGC1α/SIRT1 axis. In our study, conducted in an independent mouse model with concurrent HFD and NA administration, we observed parallel reductions in body weight gain and adipose tissue weights, thus providing additional experimental support for the metabolic regulatory role of NA. Given that energy intake was comparable across groups, whether NA attenuates HFD-induced weight gain through increased energy expenditure, enhanced adipose tissue thermogenesis, or altered lipid metabolism remains to be directly investigated.

Nevertheless, one limitation of this study should be acknowledged. In our statistical analysis, we did not account for cage-level clustering, which may lead to pseudo-replication for cage-level measurements such as food intake. However, because each group contained only two cages, the impact on the overall conclusions is likely minimal. Future studies should use cage means or mixed-effects models to properly assess cage effects.

### 4.2. Attenuating Effects of NA on Serum Lipid Profiles and Pathological Injury

Serum lipid profiling showed that mice receiving NA concurrently with an HFD had significantly lower serum TG, TC, and LDL-C levels compared with the Model group, while HDL-C levels did not differ significantly. Notably, HDL-C was also elevated in the Model group, a phenomenon attributed to the inherent lack of cholesteryl ester transfer protein (CETP) in C57BL/6J mice [22]. In humans, CETP mediates the transfer of cholesteryl esters from HDL to VLDL and LDL [23]. In its absence, HDL-associated cholesteryl esters cannot be efficiently cleared, leading to intracellular accumulation and elevated HDL-C [24]. Thus, the increased HDL-C levels in the Model group reflects severe lipid overload, and together with elevated TC, TG, and LDL-C levels confirm the establishment of systemic metabolic dysregulation following HFD feeding. NA administration significantly lowered TC, TG, and LDL-C levels, indicating effective mitigation of obesity-related dyslipidemia.

Histopathological assessment further revealed associations between NA administration and reduced pathological changes in the liver and adipose tissues. Hepatic steatosis and adipose tissue abnormalities are central pathological features of obesity-associated metabolic dysfunction [25,26]. In this study, mice receiving NA showed reduced hepatic steatosis and attenuated adipocyte hypertrophy in epididymal and perirenal adipose tissues, and these histological improvements were paralleled by a significantly lower NAFLD activity score. These organ-level observations, together with the improved lipid profiles, suggest that NA administration is associated with coordinated metabolic improvements across multiple tissues under HFD conditions. The consistency between the histopathological and serological outcomes reinforces the association between NA intervention and metabolic improvement, although the specific molecular links underlying these tissue-level correlations remain to be elucidated.

### 4.3. NA Is Associated with Altered Gut Microbiota Structure

Gut microbiota dysbiosis is widely recognized as a critical driver in the pathogenesis of obesity [27]. In the current work, 16S rRNA gene sequencing indicated that NA intervention was associated with marked alterations in the gut microbial ecosystem of HFD-fed mice.

At the α-diversity level, the Shannon and ACE indices were significantly elevated in the Model group compared with the Control, indicating altered microbial richness and evenness following HFD exposure. In the NA intervention groups, these α-diversity indices were lower than those in the Model group. It should be noted, however, that the literature regarding shifts in microbiota diversity during obesity remains inconsistent: some studies report increased diversity [28], whereas others document reduced diversity [2]. This discrepancy implies that the process of evaluating microbial health solely using α-diversity may be oversimplified, and the biological significance of directional changes in α-diversity remains unclear. A more reasonable interpretation is that HFD-induced obesity primarily causes ecological dysbiosis characterized by compositional rearrangements rather than simple gains or losses in overall diversity [29].

At the β-diversity level, PCA and PCoA analyses based on Bray–Curtis distance revealed clear separation between the Control and Model groups, confirming that the HFD dramatically reshaped global microbiota structure. Following the NA intervention, the HNA group shifted closer to the Control group in ordination space. PERMANOVA confirmed significant differences in microbial community composition among all groups, and pairwise comparisons showed that the Control group differed significantly from the Model group, and all NA intervention groups except MNA differed significantly from the Model group. These observations suggest that NA intervention is associated with a shift in community composition toward a configuration resembling that of the Control group. Whether this compositional shift reflects a direct modulatory effect of NA on the microbiota or an indirect consequence of improved host metabolism remains to be determined.

### 4.4. Bidirectional Changes in Gut Microbiota Associated with NA Intervention

Sequencing analysis showed that the relative abundance of specific microbial taxa differed between the NA intervention groups and the Model group. At the phylum level, Bacteroidota abundance was significantly higher in the NA intervention groups than in the Model group. This observation is consistent with a previous report showing that Bacteroidota levels are lower in obese individuals and increase upon weight loss [30]; the same study reported approximately 50% lower Bacteroidota levels in obese individuals compared with lean subjects, accompanied by partial restoration upon weight loss. Bacteroidota are major producers of short-chain fatty acids (SCFAs) in the gut [31]. Equipped with abundant carbohydrate-active enzymes, they ferment dietary fiber into acetate, propionate, and other SCFAs, thereby regulating energy metabolism, enhancing lipid oxidation, and reducing fat deposition [32]. Moreover, increased Bacteroidota supports intestinal barrier integrity, inhibits LPS translocation and metabolic endotoxemia, alleviates systemic low-grade inflammation, and improves insulin sensitivity [33]. An HFD typically increases the Firmicutes/Bacteroidota (F/B) ratio, which is closely linked to weight gain, insulin resistance, and chronic inflammation [34,35,36]. However, the utility of the Firmicutes/Bacteroidota (F/B) ratio as a standalone marker of dysbiosis remains controversial. Magne et al. [36] noted that the relative abundances of Firmicutes and Bacteroidetes exhibit substantial inter-individual variability among healthy populations, leading to inconsistent associations between this ratio and obesity. Brüssow [37] pointed out that dysbiosis markers such as F/B ratio changes, derived from small control cohorts, lack universal applicability and can easily misclassify normal physiological fluctuations as pathological states. Wei et al. [38] further emphasized that relying solely on a phylum-level ratio is an oversimplification that fails to capture the full complexity of gut microbial community imbalance. Therefore, the F/B ratio alone—detached from host physiological status and dietary context—is insufficient to define microbial health or dysbiosis and should be interpreted alongside microbial diversity, functional potential, and host background. In this study, HFD feeding was associated with a significantly elevated F/B ratio, and this ratio was lower in the NA intervention groups, suggesting that NA administration is accompanied by phylum-level compositional shifts under HFD conditions. The functional significance of this altered F/B ratio in the context of NA intervention remains to be clarified.

To further clarify the microbial basis of phylum-level shifts, we performed in-depth genus-level profiling. NA intervention was associated with a distinct pattern of compositional alterations, characterized by higher relative abundances of certain taxa and lower relative abundances of others.

Among enriched genera, several functionally important taxa were prominent. *Blautia* and *Oscillibacter* are major butyrate producers; as a key SCFA, butyrate stimulates GLP-1 secretion via GPR43 activation, enhancing insulin sensitivity and regulating energy intake [39,40,41]. In this study, both genera negatively correlated with LDL-C, consistent with their established metabolic benefits. Another notable group comprises bile acid-metabolizing bacteria. *Faecalibaculum* exhibits bile salt hydrolase activity and improves lipid metabolism by inhibiting the intestinal FXR-FGF15 pathway [42,43]. *Parabacteroides* also participates in bile acid metabolism and is inversely associated with BMI and fasting glucose [44,45,46,47,48]. Additionally, *Dubosiella* was significantly enriched; this genus has been shown to mitigate obesity by promoting adipose thermogenesis via upregulating Ucp1 and Prdm16 [49] and mediating the beneficial effects of exercise against non-alcoholic fatty liver disease [50]. Although *Odoribacter*, *norank_Ruminococcaceae*, and *Candidatus_Saccharimonas* are less characterized, existing evidence supports their positive roles in relieving obesity [51,52,53,54,55,56,57].

Conversely, taxa with lower relative abundance in the NA intervention groups included *Lachnoclostridium*, *Mucispirillum*, *Alistipes*, and *Bilophila*. *Lachnoclostridium* accumulates in obesity, and its abundance declines under effective anti-obesity interventions including inulin and bacterial cellulose [58,59]. *Mucispirillum* positively correlates with VLDL-C and TG [60,61]. *Alistipes* is not only associated with obesity but also carries virulence factors in certain strains and exacerbates intestinal inflammation [62,63]. *Bilophila* has been reported to synergize with HFD to worsen metabolic dysfunction [64]. These compositional shifts were accompanied by improvements in host metabolic parameters, suggesting an association between microbiota remodeling and metabolic improvement, although causality remains to be established.

Interestingly, in the NA intervention groups, we also observed enrichment of several obesity-related bacterial taxa, including *Desulfovibrio* in the LNA group, *Alistipes* in the MNA group, and *Helicobacter* in the HNA group. First, *Desulfovibrio* is a sulfate-reducing bacterium that can damage the intestinal barrier and promote lipopolysaccharide translocation by producing hydrogen sulfide (H_2_S), thereby contributing to metabolic endotoxemia [65]. The function of this genus is highly context-dependent and may be associated with host metabolic disturbances under certain conditions. It has been reported that *Desulfovibrio* is significantly increased in high-fat diet-induced obesity-prone mice and is considered one of the key genera closely linked to the obese phenotype [66]. However, some studies have also indicated potential beneficial effects on host health; for example, treatment with *Desulfovibrio vulgaris* significantly alleviated the progression of high-fat diet-induced non-alcoholic fatty liver disease in mice [67]. In the present study, neither the MNA nor the HNA group showed significant suppression of *Desulfovibrio*, yet both groups exhibited clear metabolic improvements. This observation suggests that there is no direct negative causal association between changes in *Desulfovibrio* abundance and the metabolic protective effects of NA. We speculate that the low dose of NA may be insufficient to directly inhibit *Desulfovibrio*, whereas other doses might modulate its abundance indirectly by influencing other microbial communities. Therefore, it is not rigorous to judge the improvement of obesity-related metabolism solely on the basis of changes in a single bacterial taxon; such assessments must be interpreted within the broader metabolic context.

Unlike *Desulfovibrio*, *Alistipes* exhibited a non-monotonic dose-dependent response to NA: it was significantly enriched in the MNA group but markedly decreased in the HNA group. This bidirectional fluctuation pattern suggests that changes in *Alistipes* abundance are more likely an ecological response accompanying NA intervention rather than a causal mediator, and its function is also clearly context-dependent [68]. Similarly, *Helicobacter* includes several commensal bacteria commonly found in the rodent intestine, but certain species (e.g., *H. hepaticus*, *H. bilis*) have been shown to induce chronic hepatitis, colitis, and intestinal tumors [69,70]. In metabolic research, *Helicobacter* enrichment has sometimes been associated with low-grade inflammation, but its specific role depends on the bacterial strain and host genetic background [71]. In the present study, the HNA group exhibited *Helicobacter* enrichment while simultaneously showing significant improvements in metabolic parameters. This finding indicates that *Helicobacter* enrichment did not counteract the protective effect of high-dose NA and does not necessarily reflect an intestinal pathological state. Therefore, the enrichment of *Helicobacter* in the HNA group should be regarded as a phenomenon requiring further attention, and future studies should employ metagenomics or bacterial isolation and identification for fine-resolution analysis.

### 4.5. Correlations Between Microbiota Changes and Metabolic Parameters

Correlation analysis revealed that several genera enriched after NA intervention were negatively correlated with lipid parameters: *Blautia*, *Oscillibacter*, and *norank_Ruminococcaceae* were inversely correlated with LDL-C, while *norank_Rikenellaceae* was negatively correlated with TG and perirenal fat weight. In contrast, taxa more abundant in the Model group showed positive correlations: *norank_Lachnospiraceae* was positively correlated with TC, and *Faecalimonas* was positively correlated with TG. Although these correlations do not establish causality, they suggest an association between microbiota compositional shifts and host metabolic improvement.

PICRUSt2 functional inference indicated that, following the NA intervention, the inferred abundances of amino acid metabolism and energy metabolism pathways were higher than those in the Model group, whereas the inferred carbohydrate metabolism potential was markedly lower. These inferred functional shifts were directionally consistent with the enrichment of taxa capable of short-chain fatty acid (SCFA) production, including *Blautia*, *Oscillibacter*, and *Dubosiella*, suggesting an enhanced inferred functional potential for SCFA synthesis [72,73]. Regarding the changes in carbohydrate metabolism pathways in the context of obesity, the existing literature reports model-dependent differences. Human obesity studies have shown upregulation of glycolytic and other carbohydrate metabolism pathways, which may be linked to obesity-associated inflammatory states [74]; in insulin-resistant individuals, elevated fecal carbohydrate levels, particularly monosaccharides, suggest that enhanced microbial carbohydrate metabolism may be associated with the development of obesity [75]. In animal models, interventions with slowly digestible carbohydrates have been shown to upregulate glycolysis and related pathways, accompanied by increased SCFA levels, suggesting that specific dietary interventions may improve metabolic status through modulation of carbohydrate metabolism pathways [76]. In the present study, the lower inferred carbohydrate metabolism potential observed after NA intervention differs in direction from the aforementioned reports, implying that such changes may be influenced by the type of intervention, animal model, and baseline dietary context. The inferred differences in lipid metabolism pathways paralleled the higher relative abundances of bile acid-metabolizing bacteria and the lower relative abundances of inflammation-associated taxa [77,78,79]. It should be noted that these results reflect gene-level predictions based on 16S rRNA amplicon data and do not represent actual metabolic fluxes or end-product concentrations. Therefore, whether these inferred functional changes translate into altered microbial metabolic activity remains to be determined.

Notably, all interpretations are based on correlations and functional predictions; causality has not been established. Future studies employing fecal microbiota transplantation, antibiotic depletion, gnotobiotic colonization, targeted strain validation, and microbial metabolite measurement are required to determine whether gut microbiota changes mediate the metabolic effects of NA.

## 5. Conclusions

In summary, this study demonstrates that NA administration is associated with alterations in gut microbiota composition in HFD-fed mice, accompanied by improvements in metabolic parameters. Specifically, the NA-treated groups showed higher relative abundances of taxa such as *Blautia*, *Oscillibacter*, *Faecalibaculum*, *Parabacteroides*, *Dubosiella*, and *Odoribacter* and lower relative abundances of *Lachnoclostridium*, *Mucispirillum*, *Alistipes*, *Faecalimonas*, *Bilophila*, and *norank_Lachnospiraceae*. Changes in several of these genera, including *Faecalimonas*, *Mucispirillum*, *Oscillibacter*, and *Blautia*, were correlated with improvements in TG, LDL-C, epididymal fat weight, and perirenal fat weight. These findings suggest an association between NA-induced microbiota remodeling and metabolic improvement rather than a causal relationship. Whether the observed microbial shifts contribute directly to the metabolic benefits or merely reflect improved host status remains to be determined.

This work provides preliminary association-based evidence supporting the potential application of NA as a functional food ingredient in obesity intervention and offers new perspectives for precision nutrition strategies targeting the gut microbiota. However, causality has not been established. Future investigations combining fecal microbiota transplantation and metabolomics are warranted to verify causal relationships between microbiota shifts and phenotypic improvements and to explore the translational potential of key functional strains.

## Figures and Tables

**Table 1:** PERMANOVA results for pairwise comparisons of gut microbiota composition based on Bray–Curtis distance.

Comparison	F.Model	*R* ^2^	*p*-Value
Control_vs_Model	16.96	0.68	0.008
Control_vs_LNA	15.68	0.66	0.009
Control_vs_MNA	13.57	0.63	0.008
Control_vs_HNA	19.82	0.71	0.007
Model_vs_LNA	2.61	0.25	0.018
Model_vs_MNA	1.62	0.17	0.075
Model_vs_HNA	3.41	0.30	0.009
LNA_vs_MNA	1.24	0.13	0.283
LNA_vs_HNA	2.06	0.20	0.095
MNA_vs_HNA	1.82	0.19	0.061
Between Groups	6.52	0.57	0.001

## Data Availability

The raw 16S rRNA gene sequencing data reported in this paper have been deposited in the Genome Sequence Archive (GSA) at the National Genomics Data Center (NGDC), China National Center for Bioinformation/Beijing Institute of Genomics, Chinese Academy of Sciences, under accession number CRA042397. The data are publicly accessible.

## Supplementary Materials

The following supporting information can be downloaded at: https://www.mdpi.com/article/10.3390/metabo16060399/s1, Supplementary Table S1. Composition of the normal chow diet. Supplementary Table S2. Detailed animal attrition information. Supplementary Table S3. Sequencing depth per sample after quality filtering. Supplementary Table S4. Rarefaction curves: subsampling reads at increasing depths. Supplementary Table S5. Two-way repeated-measures ANOVA results for body weight. Supplementary Figure S1. Top 25 differentially abundant bacterial genera among groups. Supplementary Figure S2. LEfSe analysis (LDA score > 3.5, *p* < 0.05).

## Abbreviations

The following abbreviations are used in this manuscript:

NA	Nervonic Acid
HFD	High-Fat Diet
NAFLD	Non-Alcoholic Fatty Liver Disease
SCFAs	Short-Chain Fatty Acids
PCA	Principal Component Analysis
PCoA	Principal Coordinate analysis
LEfSe	Linear Discriminant Analysis Effect Size
