# Supplementary figures and images for: Nervonic Acid Prevents HFD-Induced Metabolic Dysfunction and Is Associated with Gut Microbiota Remodeling
Source: Metabolites. 2026 Jun 8;16(6):399. doi: 10.3390/metabo16060399 (PMC13304234; doi:10.3390/metabo16060399)

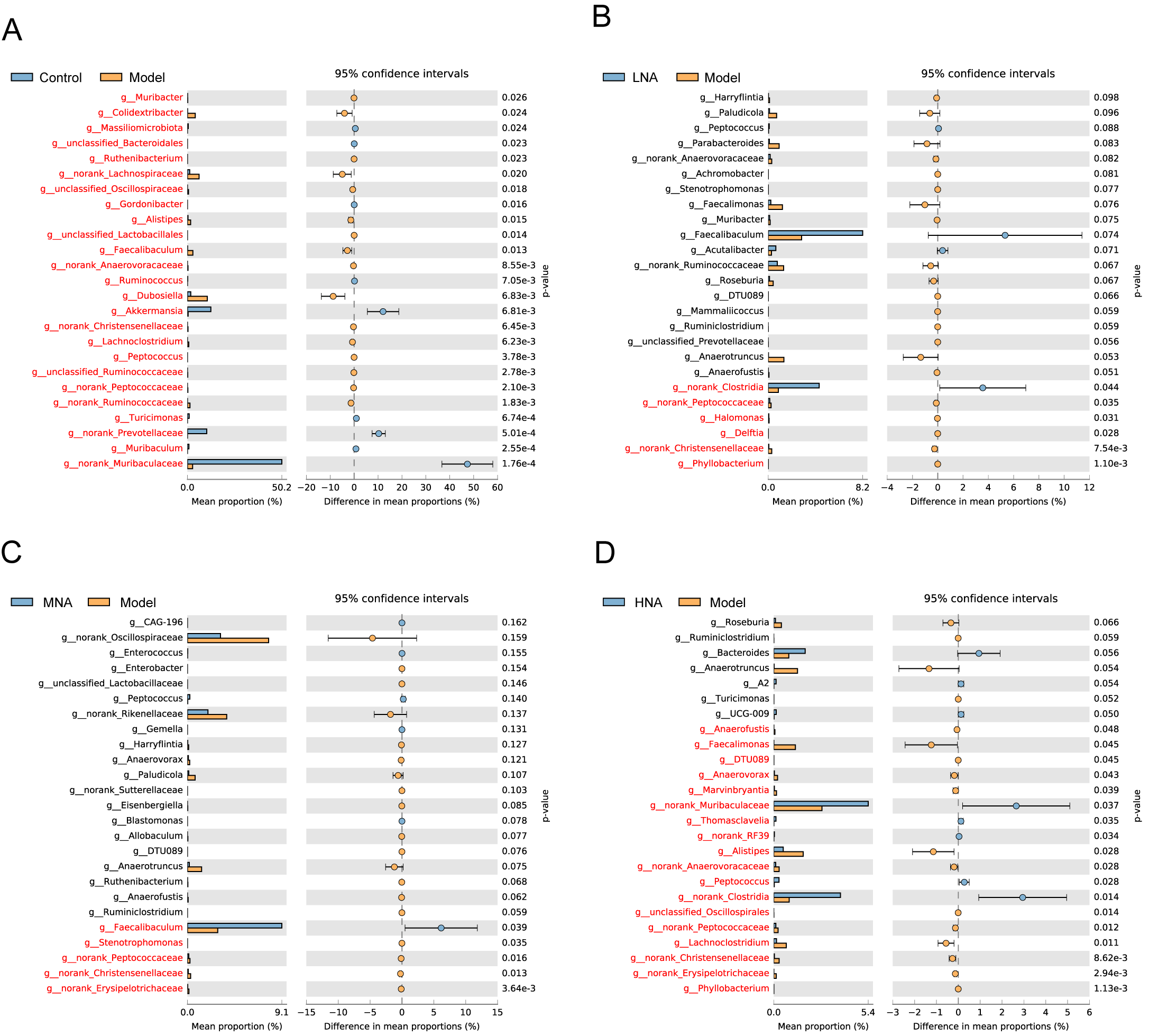

Supplement: Supplementary file 1 [file metabolites-16-00399-s001.zip › Fig S1.tif]

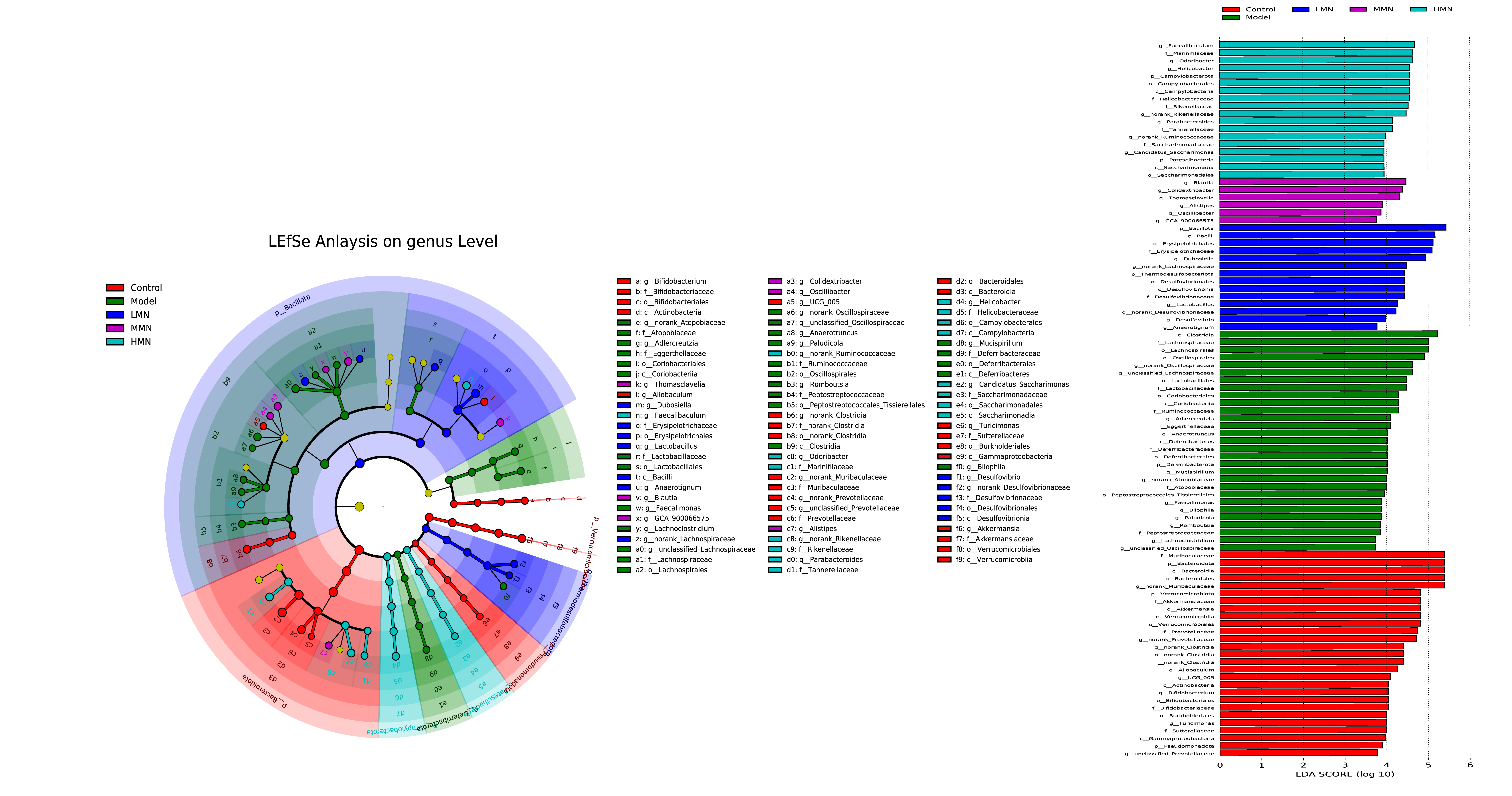

Supplement: Supplementary file 1 [file metabolites-16-00399-s001.zip › Fig S2.tif]
